# Factors underlying COVID-19 booster vaccine uptake among adults in Belgium

**DOI:** 10.1186/s13104-023-06608-4

**Published:** 2023-11-11

**Authors:** Elias Vermeiren, Joris A. F. van Loenhout, Léonore Nasiadka, Veerle Stouten, Matthieu Billuart, Izaak Van Evercooren, Lucy Catteau, Pierre Hubin

**Affiliations:** Department of Epidemiology and Public Health, Sciensano, 1050 Brussels, Belgium

**Keywords:** COVID-19 Vaccines, COVID-19 vaccine booster shot, Vaccination coverage, COVID-19 breakthrough infections, COVID-19 reinfection, Public health surveillance

## Abstract

**Objective:**

This study aimed to investigate factors influencing the uptake of first and second COVID-19 booster vaccines among adults in Belgium, particularly age, sex, region of residence and laboratory confirmed COVID-19 infection history.

**Results:**

A binomial regression model was used with having received the first or second booster as outcome and age, sex, region of residence and infection history as fixed variables. Among adults, there was generally a higher uptake to receive the first booster among older age groups compared to younger ones. Females, individuals residing in Flanders and those with no previous COVID-19 infection were more likely to receive the first booster. For the second booster, the same age trend was seen as for the first booster. Males, individuals residing in Flanders and those who tested positive for COVID-19 once after first booster were more likely to receive the second booster. Individuals with multiple positive COVID-19 tests before and after primary course or first booster were less likely to receive the subsequent booster dose compared to COVID-naïve individuals. This information could be used to guide future vaccination campaigns during a pandemic and can provide valuable insights into booster uptake patterns.

**Supplementary Information:**

The online version contains supplementary material available at 10.1186/s13104-023-06608-4.

## Introduction

In the second half of 2021, the World Health Organization (WHO) recommended administering booster vaccines against COVID-19 to adults who had already completed their primary course of COVID-19 vaccination (PC). Several studies have aimed to identify socio-demographic, socio-economic and perception-related factors associated with booster vaccine intention and hesitancy. Findings indicated that older age was positively associated with the intention to receive both first and second booster doses [1–6] while the influence of gender was inconclusive [5–7]. Higher income and education levels were associated with a higher willingness for booster vaccinations [2, 3, 6, 8]. A self-perceived risk of severe disease, considering the booster vaccine as useful and safe, and trusting in or having voted for the acting government were also associated with higher booster intention [3, 5, 6, 9, 10].

Although these studies provided insight into booster acceptance among different populations, they did not delve into actual booster uptake nor its influencing factors. Retrospective cohort studies utilizing registry-linked data could provide more comprehensive insights into the determinants of booster uptake. We identified only few articles that described registry-based studies examining the association between socio-demographic and economic factors, and COVID-19 (booster) vaccination. These studies showed a higher vaccine uptake among older individuals, females and those without prior COVID-19 infection while lower uptake was observed among socially deprived individuals and those with a migrant background [11–16]. Even though several studies looked into previous COVID-19 infection, none of the aforementioned studies have examined the potential impact of both frequency and timing of individual COVID-19 infections on booster uptake, specifically [3, 6, 9–11, 16].

In Belgium, the first booster campaign, for which the entire adult population was invited [17], took place from September 2021 until the end of February 2022. The second booster campaign started in September 2022, actively targeting individuals aged 50 and above, nursing home residents and healthcare workers [18].

Several COVID-19 waves preceded and coincided with the Belgian booster campaigns, resulting in breakthrough infections after PC. These individuals may harbor scepticism about vaccine effectiveness compared to those infected before vaccination or uninfected individuals [3, 6]. This nation-wide, registry-based, cohort study aimed to identify which factors, including laboratory-confirmed COVID-19 infection history and demographic characteristics, are associated with the uptake of first and second COVID-19 booster vaccines in Belgium.

## Methods

### Data sources

Two databases were used in this analysis: (1) the COVID-19 national vaccination registry (VaccinNet +) which contains demographic information (age, sex and region of residence) and COVID-19 vaccination details (vaccine brand, date of administration and number of doses received), and (2) the COVID-19 Health Data test database which contains individual COVID-19 test data (PCR and antigen test results and testing dates). These databases were linked using the unique Belgian national registry number. Data were pseudonymised before conducting the analyses.

### Study population

All adults (≥ 18 years) who had PC in Belgium—2 doses of Comirnaty^®^ (Pfizer/BioNtech), Spikevax^®^ (Moderna), Vaxzevria^®^ (AstraZeneca-Oxford) or 1 dose of Jcovden^®^ (Johnson & Johnson)—were included in the analyses focusing on first booster uptake. Persons with incomplete PC (only one dose over two) or a heterologous PC (PC completed using doses of 2 different COVID-19 vaccines, often found to be an erroneous registration) were excluded from the analyses. For analyses regarding the second booster uptake, those who received one booster dose after a PC and were aged 50 years or older were included (as only persons over 50 were actively invited for a second booster during the Belgian vaccination campaign). Only individuals who received a booster with Comirnaty^®^ (Pfizer/BioNtech) or Spikevax^®^ (Moderna) were included in the analyses. First boosters administered until March 1st 2022 and second boosters until January 31st were considered. We only included persons alive on the 31st of January 2023.

Infection history of individuals was based on their laboratory-confirmed COVID-19 infection status, utilizing PCR and antigenic COVID-19 tests. This was considered relative to the last preceding COVID-19 vaccination. As shown in Table 1, five levels of infection history were defined. For simplicity, we will refer to this as “infection history” moving forward.

**Table 1:** Discretized laboratory-confirmed COVID-19 infection history levels for first and second booster uptake

Level	Infection history	
	First booster	Second booster
COVID-naïve	No infection before first booster or end date^a^	No infection before second booster or end date^b^
At least 1 infection before	At least 1 infection before PC	At least 1 infection before 1st booster
1 infection after	1 infection between PC and 1st booster or end date^a^	1 infection between 1st booster and 2nd booster or end date^b^
> 1 infection after	More than 1 infection between PC and 1st booster or end date^a^	More than 1 infection between 1st booster and 2nd booster or end date^b^
> 1 infection before and after	More than one infection until first booster or end date^a^, before as well as after PC	More than one infection until end date^b^, before as well as after 1st booster

*PC* primary course completion

^a^March 1st 2022, end of the first booster campaign

^b^January 31st 2023, end of the second booster campaign

### Statistical analysis and measures

We fitted a logistic regression model, assuming a quasi-binomial data distribution (number of boosted over number of people who received previous vaccination), with having received a first or a second booster as outcome (number of successes) and with age, sex, region of residence and infection history as categorical fixed effects. Additionally, a sensitivity analysis was performed based on stratification by age, by fitting logistic regression models with the same fixed effects apart from age. The model coefficients were used to calculate the adjusted odds ratio (aOR). All statistical calculations were performed in R (version 4.1.3).

## Results

### Study population

The study population comprised 7 857 113 individuals, of whom 51.42% were female. The mean age was 50.6 ± 18.8 years. Among those who received the PC, 82.55% received the first booster and 46.57% received the second booster. Among the first booster recipients, 84.00% were COVID-naïve, 8.00% had at least one prior infection before PC and 7.68% had one infection after PC. Less than 1% of the study population had multiple infections before and after, or only after PC. Among second booster recipients, 74.16% were COVID-naïve, 11.99% had at least one infection before the first booster and 12.73% had one infection after the first booster. Less than 2% had multiple infections before and after or only after the first booster (Table 2).

**Table 2:** First and second booster uptake by individual characteristics at 31st of January 2023 in Belgium

Variable	Level	Primary Course N (%)	First Booster N (%)	Second Booster N (%)
Overall	All	7 857 113 (100.00)	6 485 659 (82.55)^a^	3 658 711 (46.57)^a^
Sex	Female	4 039 983 (51.42)	3 355 007 (51.73)	1 930 865 (52.77)
	Male	3 817 130 (48.58)	3 130 652 (48.27)	1 727 846 (47.23)
Age group	18–24	733 453 (9.33)	508 787 (7.84)	131 218 (3.59)
	25–34	1 160 075 (14.76)	823 290 (12.69)	261 200 (7.14)
	35–44	1 233 228 (15.70)	927 832 (14.31)	343 136 (9.38)
	45–54	1 327 719 (16.90)	1 096 158 (16.90)	543 324 (14.85)
	55–64	1 394 302 (17.75)	1 243 104 (19.17)	829 106 (22.66)
	65–74	1 097 031 (13.96)	1 027 869 (15.85)	819 676 (22.40)
	75–84	648 618 (8.26)	610 983 (9.42)	517 445 (14.14)
	85 +	262 687 (3.34)	247 636 (3.82)	213 606 (5.84)
Region of residence	Brussels	657 983 (8.37)	412 677 (6.36)	160 176 (4.38)
	Flanders	4 854 958 (61.79)	4 284 468 (66.06)	2 800 191 (76.53)
	Wallonia	2 344 172 (29.84)	1 788 514 (27.58)	698 344 (19.09)
Infection history	COVID-naïve	NA	5 447 844 (84.00)^b^	2 713 334 (74.16)^c^
	At least 1 infect. before	NA	519 082 (8.00)^b^	438 519 (11.99)^c^
	1 infect. after	NA	498 187 (7.68)^b^	465 636 (12.73)^c^
	> 1 infect. after	NA	690 (0.01)^b^	4 997 (0.14)^c^
	> 1 infect. before and after	NA	19 856 (0.31)^b^	36 225 (0.99)^c^

The percentages show the distribution per outcome for each vaccination status at the 31st of January 2023 among Belgian residents

*Infect*. laboratory-confirmed COVID-19 infection, *NA* not applicable

^a^The percentage for the ‘Overall’ numbers show the coverage of uptake over number of persons who received primary course.

^b^Infection history relative to PC

^c^Infection history relative to first booster

### Factors associated with booster uptake

When investigating the influence of infection history on the first booster uptake, we saw that individuals with no registered COVID-19 infection were more likely to have received a first booster (Table 3), this group was used as reference. Those with at least one infection before PC, had a lower likelihood of getting the first booster [aOR = 0.69, 95%CI (0.63;0.75)]. Similarly, individuals with multiple infections before and after PC [aOR = 0.17, 95%CI (0.13;0.21)] or with only one infection after PC [aOR = 0.33, 95%CI (0.31;0.36)] had a lower likelihood of receiving the first booster. The least likely to receive the first booster were individuals with more than one infection after PC but none before [aOR = 0.11, 95%CI (0.03;0.37)].

**Table 3:** Adjusted odds ratio for regression model coefficients for the first and second booster uptake

Variable	Level	aOR [95% CI]	
		First booster	Second booster
Sex	Female	*Ref*	*Ref*
	Male	0.93 [0.88; 0.98]	1.07 [1.04; 1.11]
Age group	18–24	*Ref*	NA
	25–34	1.13 [1.04; 1.23]	NA
	35–44	1.41 [1.29; 1.54]	NA
	45–54^a^ or 50–54^b^	2.09 [1.91; 2.29]	*Ref*
	55–64	3.31 [3.00; 3.64]	1.71 [1.63; 1.79]
	65–74	5.56 [4.94; 6.27]	3.56 [3.39; 3.75]
	75–84	5.69 [4.91; 6.59]	4.91 [4.61; 5.23]
	85 +	5.23 [4.24; 6.45]	5.63 [5.14; 6.16]
Region of residence	Flanders	*Ref*	*Ref*
	Brussels	0.23 [0.21; 0.25]	0.29 [0.27; 0.31]
	Wallonia	0.41 [0.39; 0.44]	0.31 [0.30; 0.32]
Infection history	COVID-naïve	*Ref*	*Ref*
	At least 1 infect. before	0.69 [0.63; 0.75]^c^	0.87 [0.82; 0.92]^d^
	1 infect. after	0.33 [0.31; 0.36]^c^	1.09 [1.03; 1.15]^d^
	> 1 infect. after	0.11 [0.03; 0.37]^c^	0.46 [0.30; 0.71]^d^
	> 1 infect. before & after	0.17 [0.13; 0.21]^c^	0.82 [0.69; 0.98]^d^

*NA* not applicable, *Ref.* level used as reference, *infect*. laboratory-confirmed COVID-19 infection

^a^For analysis first booster uptake (population ≥ 18 years)

^b^For analysis second booster uptake (population ≥ 50 years)

^c^Infection history relative to PC

^d^Infection history relative to first booster

Compared to the youngest age group (18–24 years), older age groups showed higher likelihood of receiving the first booster, with the 75–84 age group being the most likely [aOR = 5.69, 95%CI (4.91;6.59)]. Men had a slightly lower likelihood to receive the first booster compared to women. Residents of Flanders had the highest odds of receiving a first booster compared to Wallonia and Brussels.

Compared to COVID-naïve individuals, those with one infection after first booster were slightly more likely to receive the second booster [1 infection after first booster aOR = 1.09, 95%CI (1.03;1.15)]. However, individuals with other infection history patterns were consistently less likely to receive one, compared to COVID-naïve individuals [at least 1 infect. before aOR = 0.87, 95%CI (0.82;0.92); multiple infections before and after first booster aOR = 0.82, 95%CI (0.69;0.98); more than 1 infection after first booster aOR = 0.46, 95%CI (0.30;0.71)].

Compared to the youngest age group (50–54 years), higher age groups were more likely to receive the second booster, with the over 85 age group having the highest likelihood [aOR = 5.63, 95%CI (5.14;6.16)]. Men had a slightly higher likelihood to get the second booster compared to women. Individuals residing in Flanders had the highest likelihood of receiving the second booster compared to Wallonia and Brussels (Table 3).

We performed a sensitivity analysis, in which we explored a potential interaction between infection history and age by refitting the models stratified by age group. Overall, the results resembled closely the results of the main analyses (Additional file 1: Fig. S1).

## Discussion

Individuals who had been previously infected were less likely to have received a first booster than COVID-naïve individuals. This effect of infection history on first booster uptake was also influenced by the timing and number of infections, with a decrease in uptake observed when the number of infections increases, especially after PC. The COVID-19 vaccines were initially portrayed as the pandemic-terminating solution, which may have caused distrust and disbelief in vaccine effectiveness within persons who got infected after PC. Distrust in vaccine effectiveness and safety were deemed drivers in booster hesitancy, demonstrated by Della Polla et al., Bennett et al., Sprengholz et al. and Waterschoot et al. [3, 5, 9, 10]. Belgium encountered several COVID-19 waves (Delta and Omicron) during the first booster campaign, causing a rise in breakthrough infections. Since an individual had to be at least two weeks COVID-19-free to be eligible for a booster dose, the occurrence of infections too close to the proposed booster administration date, could have influenced persons’ willingness and ability to receive the first booster as well.

A small difference was observed in the effect of infection history on the second booster uptake compared to the first booster, with individuals having had one infection after first booster being slightly more likely to have received the second booster than COVID-naïve individuals. This could be partially attributed to the shift in timing of the infection history (relative to first booster instead of PC), but also to changes in testing strategies and non-pharmaceutical interventions during booster campaigns. During the first booster campaign, Belgium was still in a “crisis mode” with various restrictions and a more elaborate testing strategy. However, as 2022 progressed, the testing capacity and test prescriptions decreased, resulting in a decrease in registered PCR tests. This led to an underestimation of individuals’ infection history relative to the first booster uptake, which causes a vast overestimation of COVID-naïve individuals. The COVID-naïve group was used as reference for infection history in both uptake models, considering its heterogenous nature in the second booster uptake model, it will influence the model outcome directly. Individuals with all other infection history patterns were consistently less likely to have received the second booster, following the trends observed in the first booster uptake. Nevertheless, given the aOR, infection history had a more sizable effect on vaccine uptake for the first booster campaign compared to the second. Bennett et al. and Della Polla et al. reported on the possible effect of previous COVID-19 infection on first and second booster intention respectively, based on self-reported surveys with limited study samples, but the results were not significant [9, 10]. A study by Hansen et al. showed a significant negative effect of COVID-19 infection on first booster uptake [11], which is in line with our study results.

We observed an increasing likelihood of first and second COVID-19 booster uptake with increasing age, consistent with studies on booster acceptance [1–6, 11, 12]. While females were slightly more likely of having received the first booster, the opposite was observed for the second booster. Nevertheless, the difference between genders was minimal. Studies investigating booster willingness showed as well conflicting results concerning sex [5–7]. Regional differences in booster uptake could possibly be explained partially by socio-economic factors and migration background which can influence vaccine hesitancy [2, 8, 9, 11, 12]. The Belgian regional effect was also demonstrated by a previous study concerning PC uptake in Belgium [13].

## Conclusion

Older age, residing in Flanders (compared to the other Belgian regions) were positively associated with both first and second booster uptake. Having had multiple confirmed COVID-19 infections before or after preceding vaccination, were negatively associated with the first and second booster uptake. These findings highlight the potential influence of being previously infected with COVID-19 on individuals’ booster vaccine uptake. This information could give a better estimation of expected booster vaccine uptake in future booster vaccine campaigns.

## Limitations

We acknowledge three main limitations. Firstly, the infection history data is limited due to the evolving testing strategy. The incomplete testing of symptomatic individuals and the absence of antigenic self-test results, particularly during the period in between the first and second booster (start of Omicron dominance), resulted in an overestimation of COVID-naïve individuals. However, we believe that this limitation has minimal impact on the overall direction of our estimates given the repeated negative effect of having received multiple infections on booster uptake. Secondly, we cannot asses the influence of individuals perception regarding booster vaccines. Several studies have shown the inherent link between perception, regarding politics, religion, vaccine safety and effectiveness, risk of (severe) disease and peer behaviour, and booster hesitancy [3, 5, 6, 10]. Our database does not contain any perception data, hence we cannot link observational data with opinionated data. Thirdly, our model included basic demographic factors and infection history as predictors, but other individual-level factors can influence booster uptake as well, as shown in other studies [11–14, 16]. Variables such as household income, migration background, level of social deprivation, profession (healthcare or non-healthcare) and underlying illnesses, will be considered in future analyses to provide a more comprehensive understanding of booster uptake.

### Supplementary Information

**Additional file 1:** **Figure S1**. Comparison of drivers booster uptake in general models and age group-stratified models. **A** Odds ratio for the general model for first booster uptake. **B** Odds ratio for the general model for second booster uptake. **C** Odds ratio for the age group-stratified model for first booster uptake. CI is not shown for ‘>1 infect. after’ due to out of bounds. **D** Odds ratio for the age group-stratified model for second booster uptake

## Data Availability

As the LINK-VACC project is not an open-access platform, the individual level data are only available to researchers working on the project. However, general descriptive statistics from the registries used in LINK-VACC are available from https://epistat.sciensano.be/covid/. This includes *inter alia* the number of confirmed cases by date, age, sex and province or the number of administered vaccines by date, region, age, sex, brand and dose.

## Abbreviations

PC: Completed COVID-19 primary course vaccination
aOR: Adjusted odds ratio
