# Supplementary figures and images for: Factors underlying COVID-19 booster vaccine uptake among adults in Belgium
Source: BMC Res Notes. 2023 Nov 11;16:328. doi: 10.1186/s13104-023-06608-4 (PMC10640742; doi:10.1186/s13104-023-06608-4)

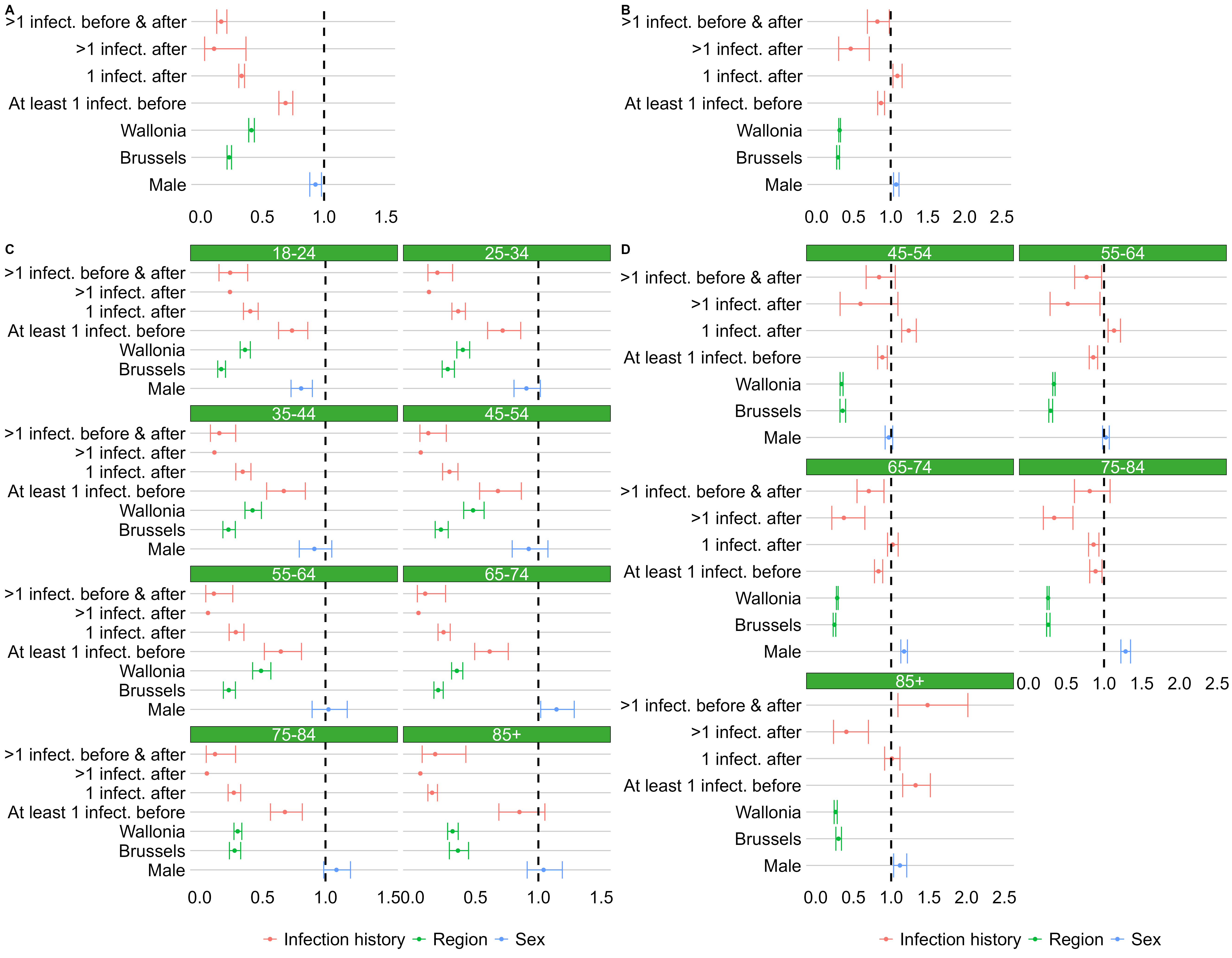

Supplement: Supplementary file 1 — Additional file 1: Figure S1. Comparison of drivers booster uptake in general models and age group-stratified models. A Odds ratio for the general model for first booster uptake. B Odds ratio for the general model for second booster uptake. C Odds ratio for the age group-stratified model for first booster uptake. CI is not shown for ‘>1 infect. after’ due to out of bounds. D Odds ratio for the age group-stratified model for second booster uptake [file 13104_2023_6608_MOESM1_ESM.jpg]
